# Enhanced Activation of the S1PR2-IL-1β-Src-BDNF-TrkB Pathway Mediates Neuroinflammation in the Hippocampus and Cognitive Impairment in Hyperammonemic Rats

**DOI:** 10.3390/ijms242417251

**Published:** 2023-12-08

**Authors:** María Sancho-Alonso, Yaiza M. Arenas, Paula Izquierdo-Altarejos, Mar Martinez-Garcia, Marta Llansola, Vicente Felipo

**Affiliations:** 1 Laboratory of Neurobiology, Centro de Investigación Príncipe Felipe, 46012 Valencia, Spain; maria.sancho@iibb.csic.es (M.S.-A.); ymarenas@cipf.es (Y.M.A.); pizquierdo@cipf.es (P.I.-A.); mmartinezg@cipf.es (M.M.-G.); vfelipo@cipf.es (V.F.); 2 Institute of Biomedical Research of Barcelona (IIBB), Spanish National Research Council (CSIC), 08036 Barcelona, Spain; 3 Systems Neuropharmacology Research Group, Institut d’Investigacions Biomèdiques August Pi i Sunyer (IDIBAPS), 08036 Barcelona, Spain; 4 Biomedical Research Networking Center for Mental Health (CIBERSAM), Institute of Health Carlos III (ISCIII), 28029 Madrid, Spain

**Keywords:** hepatic encephalopathy, cognitive impairment, neuroinflammation, sphingosine-1-phosphate, glutamate receptor, BDNF

## Abstract

Hyperammonemia contributes to hepatic encephalopathy. In hyperammonemic rats, cognitive function is impaired by altered glutamatergic neurotransmission induced by neuroinflammation. The underlying mechanisms remain unclear. Enhanced sphingosine-1-phosphate receptor 2 (S1PR2) activation in the cerebellum of hyperammonemic rats contributes to neuroinflammation. in In hyperammonemic rats, we assessed if blocking S1PR2 reduced hippocampal neuroinflammation and reversed cognitive impairment and if the signaling pathways were involved. S1PR2 was blocked with intracerebral JTE-013, and cognitive function was evaluated. The signaling pathways inducing neuroinflammation and altered glutamate receptors were analyzed in hippocampal slices. JTE-013 improved cognitive function in the hyperammonemic rats, and hyperammonemia increased S1P. This increased IL-1β, which enhanced Src activity, increased CCL2, activated microglia and increased the membrane expression of the NMDA receptor subunit GLUN2B. This increased p38-MAPK activity, which altered the membrane expression of AMPA receptor subunits and increased BDNF, which activated the TrkB → PI3K → Akt → CREB pathway, inducing sustained neuroinflammation. This report unveils key pathways involved in the induction and maintenance of neuroinflammation in the hippocampus of hyperammonemic rats and supports S1PR2 as a therapeutic target for cognitive impairment.

## 1. Introduction

Chronic hyperammonemia is a main contributor to the cognitive and motor impairment in cirrhotic patients with minimal hepatic encephalopathy (MHE) or clinical HE [1,2,3,4,5], which impair quality of life [6,7,8,9].

Cognitive impairment in patients with MHE is associated with structural and functional connectivity alterations in hippocampus [10,11,12].

Also, in animal models, the mechanisms by which hyperammonemia impairs cognitive function involve altered glutamatergic neurotransmission in hippocampus which, in turn, is a consequence of neuroinflammation [2,3,13,14,15,16,17].

Hyperammonemia induces peripheral inflammation, which induces neuroinflammation in the hippocampus [16]. This neuroinflammation is associated with the activation of microglia and astrocytes and increased levels of the pro-inflammatory cytokines, such as IL-1β and TNFα, in the hippocampus [18,19]. IL-1β and TNFα activate their receptors in hippocampal neurons leading to altered membrane expression of AMPA and NMDA receptors [18,19,20,21,22,23], which, in turn leads to impaired cognitive function [18,19,24,25].

In rats with chronic hyperammonemia, cognitive function may be restored by reducing neuroinflammation with sulphorafane, peripheral anti-TNFα or extracellular cGMP [14,16,18]. This indicates that reducing neuroinflammation may also be useful to improve cognitive function in cirrhotic patients with MHE. To design treatments to reduce neuroinflammation in the hippocampus in chronic hyperammonemia and MHE it is necessary to understand the molecular mechanisms involved in the induction and maintenance of microglia and astrocytes activation of neuroinflammation.

Taoro-Gonzalez et al. [19] showed that increased IL-1β levels play a main role in altering glutamatergic neurotransmission in the hippocampus and in inducing cognitive impairment, which may be reversed by blocking the IL-1 receptor. However, how hyperammonemia increases IL-1β in the hippocampus remains unclear. One of the aims of this work was to identify the underlying mechanism.

McMillin et al. [26,27] reported that neuronal CCL2 is upregulated and contributes to microglia activation and neurological decline in mice with HE induced by azoxymethane [26] and that enhanced activation of the sphingosine-1-phosphate receptor 2 (S1PR2) promotes neuroinflammation in the cerebral cortex of these mice [27,28,29,30]. We recently showed that enhanced activation of S1PR2 also contributes to neuroinflammation in the cerebellum and to motor incoordination in hyperammonemic rats [31]. Enhanced activation of S1PR2 increases CCL2 levels leading to microglia activation, increased BDNF levels and activation of TrkB, which enhances GABAergic neurotransmission in the cerebellum, leading to motor incoordination. Blocking S1PR2 with the antagonist JTE-013 reverses neuroinflammation and restores motor coordination in hyperammonemic rats [31]. We hypothesized that enhanced activation of S1PR2 would also contribute to the increase in IL-1β and to induction of neuroinflammation in the hippocampus of hyperammonemic rats. In addition, we hypothesized that the enhanced activation of S1PR2 would contribute to glial activation, neuroinflammation in the hippocampus, altered membrane expression of AMPA and NMDA receptors, and cognitive impairment in hyperammonemic rats and that blocking S1PR2 with JTE-013 would reverse all of these effects.

The aims of this work were to (1) assess if blocking S1PR2 in vivo with the antagonist JTE-013 reverses cognitive impairment in hyperammonemic rats; (2) assess if this is associated with a reversal of alterations in the membrane expression of AMPA and NMDA receptors in the hippocampus; (3) assess if this is associated with a reversal of glial activation and neuroinflammation; (4) identify pathways by which enhanced S1PR2 activation induces neuroinflammation in the hippocampus of hyperammonemic rats.

## 2. Results 

**Figure 1:**
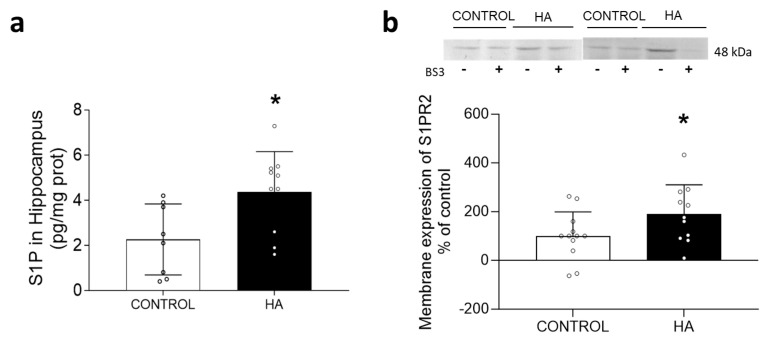
Hyperammonemia increased the S1P content and membrane expression of S1PR2 in the hippocampus. The content of the S1P in hippocampal slices was quantified using LC-MS (**a**). Values are the means ± SD of 8 control and 10 hyperammonemic rats. Membrane expression of S1PR2 in hippocampal slices (**b**). Values are the means ± SD of 11–12 rats per group. The data were analyzed using a Student’s *t*-test. Values significantly different from the control rats are indicated by asterisks, where * *p* < 0.05.

The S1P levels increased to 4.4 ± 1.8 pg/mg protein (*p* < 0.05) in the hippocampal slices of the hyperammonemic rats compared with 2.3 ± 1.6 pg/mg protein in the control rats (Figure 1a). Moreover, the membrane expression of its receptor, S1PR2, also increased (*p* < 0.05) to 190 ± 120% in the control rats (Figure 1b). The increases in both the S1P content and S1PR2 membrane expression resulted in enhanced activation of S1PR2 in the hippocampus of the hyperammonemic rats.

We then assessed whether enhanced SIPR2 receptor activation contributes to impaired cognitive function in hyperammonemic rats by blocking S1PR2 with the specific antagonist JTE-013.

### 2.1. Blocking S1PR2 in Vivo with JTE-013 Reverses Cognitive Impairment in Hyperammonemic Rats

**Figure 2:**
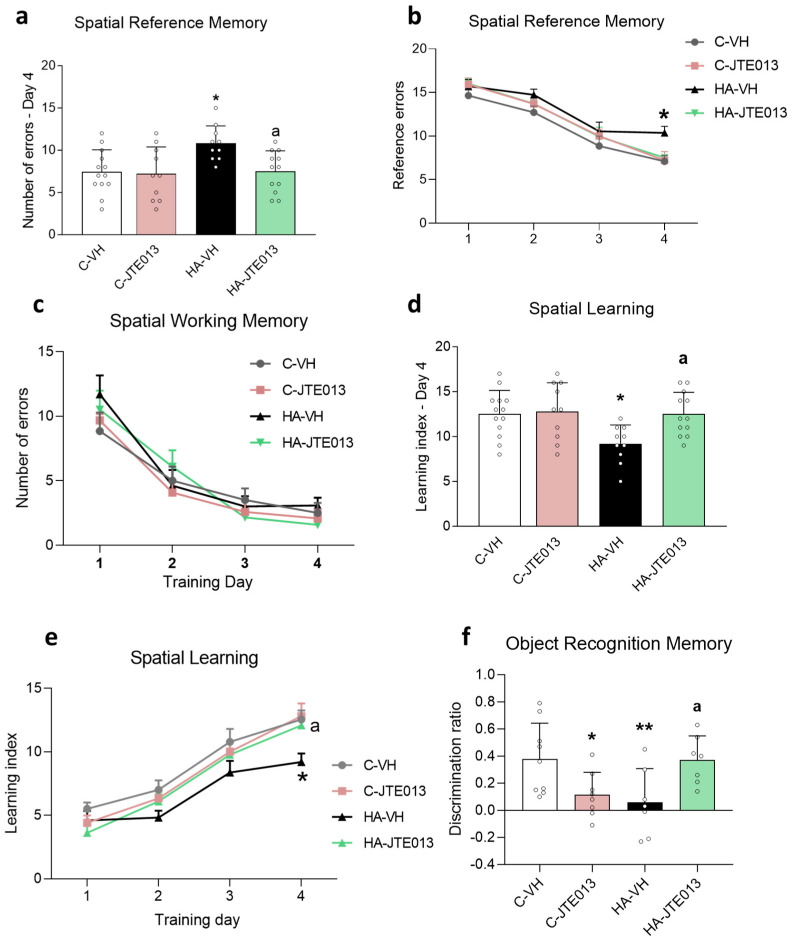
Hyperammonemia impaired spatial reference memory, learning index and novel object recognition. JTE013 treatment reversed these alterations. Spatial and working learning and memory were assessed in the control (C) and hyperammonemic (HA) rats treated with vehicle or JTE013 in the radial maze: spatial reference errors (**a**,**b**); spatial working errors (**c**); spatial learning index (**d**,**e**); discrimination ratio in the novel object recognition task (**f**). The values are the means ± SD of 10–13 rats per group for the radial maze and 7–9 rats per group for the object recognition task. The data were analyzed using two-way ANOVA and Fisher’s LSD multiple comparisons post hoc test (**a**,**d**,**f**), as well as two-way ANOVA with repeated measures with a Bonferroni post hoc test (**b**,**c**,**e**). Values significantly different from the control rats and HA rats are indicated by asterisks and a, respectively, where * *p* < 0.05, ** *p* < 0.01, and a *p* < 0.05.

Hyperammonemic rats showed impaired learning and memory, as assessed in the radial maze (Figure 2). Hyperammonemia increased the reference errors at day 4 (Figure 2a,b), but it did not affect working memory errors (Figure 2c) and reduced the learning index at day 4 (Figure 2d,e). Treatment with JTE-013 reversed the impairment in the reference memory and learning index (Figure 2a–e).

Hyperammonemia also impaired novel object recognition memory (Figure 2f), which was also restored by treatment with JTE-013.

These data show that blocking S1PR2 with JTE-013 reversed the cognitive impairment in hyperammonemic rats. We then analyzed the molecular mechanisms involved. We have shown that the ex vivo treatment of freshly isolated cerebellar slices from hyperammonemic rats with JTE-013 reproduces the effects and mechanisms observed in vivo for induction of neuroinflammation, glial activation and alterations in neurotransmission [31]. Also, for the hippocampus, the treatment of freshly isolated hippocampal slices from hyperammonemic rats with an antagonist of the IL-1 receptor reproduces the effects and mechanisms observed in vivo [19]. We, therefore, used an ex vivo system consisting of freshly isolated hippocampal slices from the control and hyperammonemic rats to study the mechanisms by which blocking S1PR2 with JTE-013 improves cognitive function in hyperammonemic rats.

We have shown that this cognitive impairment is due to the altered membrane expression of AMPA and NMDA receptors [18,19,32]. We, therefore, assessed if treatment with JTE-013 reverses these alterations in the membrane expression of glutamate receptors in the hippocampus of hyperammonemic rats.

### 2.2. Blocking S1PR2 Ex Vivo with JTE-013 Reverses Altered Membrane Expression of AMPA and NMDA Receptors in Hippocampal Slices from Hyperammonemic Rats

**Figure 3:**
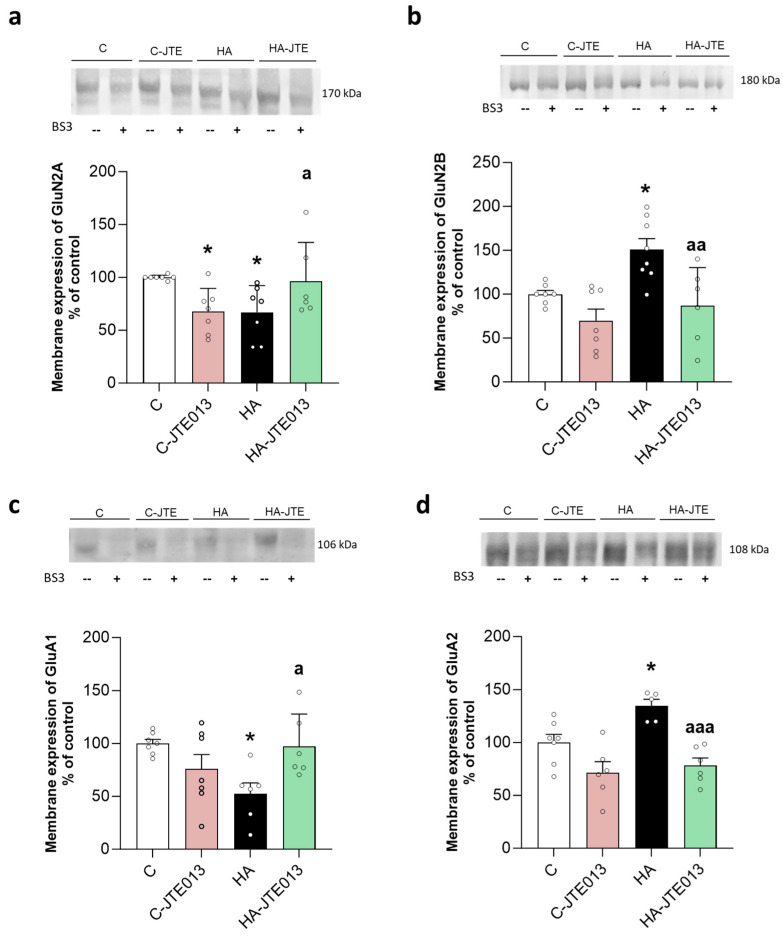
Hyperammonemia altered the membrane expression of NMDA and AMPA receptor subunits in the hippocampus. JTE013 treatment reversed these alterations. The membrane expressions of GluN2A (**a**) GluN2B (**b**), GluA1 (**c**), and GluA2 (**d**) were analyzed using the BS3 cross-linker procedure in hippocampal slices from control (C) and hyperammonemic (HA) rats treated with vehicle or JTE-013 to block S1PR2. The values are the means ± SD of 6–8 experiments with duplicate samples in each one. The data were analyzed using two-way ANOVA followed by Fisher’s LSD (**a**) or Tukey’s (**b**–**d**) post hoc test. Values significantly different from the control rats are indicated by asterisks, where * *p* < 0.05, and from the hyperammonemic rats by “a”, where a *p* < 0.05, aa *p* < 0.01, and aaa *p* < 0.001.

Hyperammonemia reduces the membrane expression of the GluN2A subunit of NMDA receptors (Figure 3a) and increases that of GluN2B (Figure 3b). Concerning the AMPA receptor subunits, hyperammonemia reduces the membrane expression of GluA1 (Figure 3c) and increases that of GluA2 (Figure 3d). Treatment with JTE-013 reverses the changes induced by hyperammonemia in the membrane expression of all of these subunits, returning them to normal values (Figure 3a–d). The normalization of the membrane expression of the NMDA and AMPA receptors contributes to the reversal of the cognitive impairment by JTE-013.

The altered membrane expression of AMPA and NMDA receptors in the hippocampus of hyperammonemic rats is a consequence of neuroinflammation, glial activation and increased levels of IL-1β [18,19,32]. We, therefore, assessed if JTE-013 reverses glial activation, neuroinflammation and IL-1β levels in the hippocampus of hyperammonemic rats and some of the underlying mechanisms.

### 2.3. Blocking S1PR2 Ex Vivo with JTE-013 Reverses Microglia and Astrocytes Activation and the Increase in IL-1β in Hippocampal Slices from Hyperammonemic Rats

**Figure 4:**
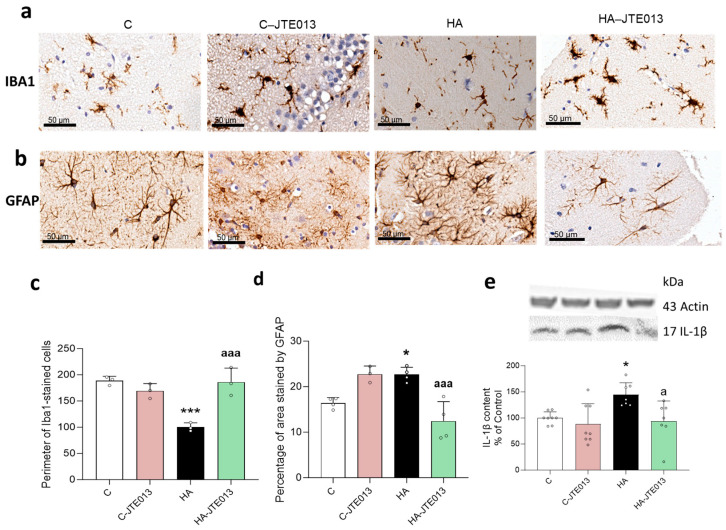
Blocking S1PR2 with JTE013 reduced the microglia and astrocyte activation and normalized the content of IL-1β in the hippocampus of the hyperammonemic rats. Immunohistochemistry was performed on hippocampal slices from control and hyperammonemic rats treated or not with JTE-013 using antibodies against Iba1 (**a**,**c**) and GFAP (**b**,**d**). To analyze the microglia activation, the perimeter of cell was measured. The GFAP-stained area was measured as an indicator of astrocytes activation. The values are the means ± SD of 3–4 experiments with duplicate samples in each one. The total content of IL-1β (**e**) in the slices of hippocampus from the control and hyperammonemic rats treated or not with JTE-013 was analyzed using Western blot. The values are the means ± SD of 7–8 experiments with duplicate samples in each one. The data were analyzed using two-way ANOVA followed by Tukey’s post hoc test. Values significantly different from the control rats are indicated by asterisks, where * *p* < 0.05 and *** *p* < 0.001, and from the hyperammonemic rats by “a”, where a *p* < 0.05 and aaa *p* < 0.001.

Chronic hyperammonemia increased microglia activation in the hippocampus, which is reflected in a reduction in the perimeter of microglial cells stained with Iba1 (100 ± 8 μm) compared to the control rats (189 ± 8 μm) (*p* < 0.001) (Figure 4a,c). The ex vivo treatment of hippocampal slices from the hyperammonemic rats with JTE-013 to block S1PR2 completely reversed the microglia activation, returning the perimeter of the microglia to normal values (187 ± 38 μm, *p* < 0.05) (Figure 4a,c).

Hyperammonemia also increased the activation of astrocytes, reflected in an increase in the area covered by the anti-GFAP staining (23 ± 1.6%; *p* < 0.05) compared to the control rats (16 ± 1.2) (Figure 4b,d), while JTE-013 reversed the astrocytes activation (12 ± 4.3%, *p* < 0.01) (Figure 4b,d).

Hyperammonemia increased the IL-1β content in the hippocampus (139 ± 26%, *p* < 0.01), while blocking S1PR2 with JTE-013 completely reversed the increase in IL-1β (102 ± 17%; *p* < 0.05) (Figure 4e). 

### 2.4. Increaseing IL-1β Enhances Activation of IL-1 Receptor, Which Activates Src, Increasing CCL2 Levels in Hippocampus of Hyperammonemic Rats, Which Is Reversed by Blocking S1PR2 or the IL-1 Receptor and by Inhibiting Src

**Figure 5:**
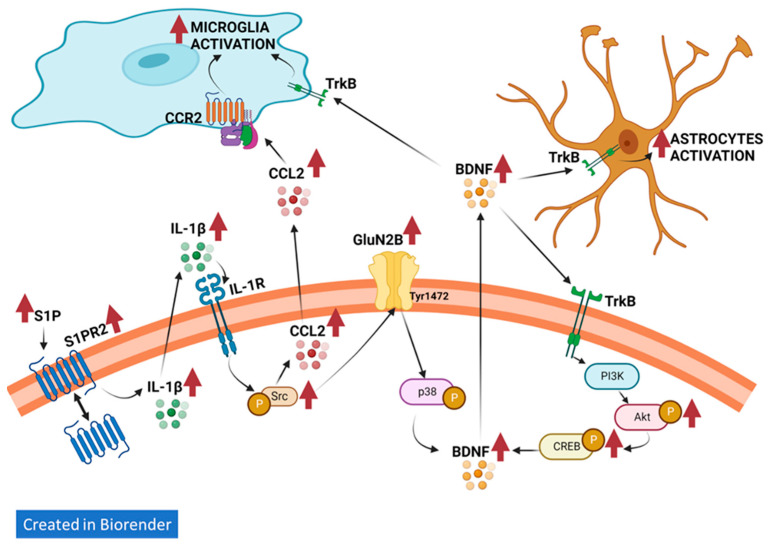
Pathways involved in the neuroinflammatory process in the hippocampus of hyperammonemic rats. Hyperammonemia increases S1PR2 activation in the hippocampus by increasing the level of S1P and the membrane expression of S1PR2. This increases IL-1β in neurons and the activation of its receptor, IL-1R, which enhances Src activity leading to increased phosphorylation and membrane expression of the GLUN2B subunit of NMDA receptors. This leads to increased activity of p38 MAP kinase and altered membrane expression of the GluA1 and GluA2 subunits of AMPA receptors, which results in cognitive impairment. Blocking S1PR2 with JTE-013 in vivo restores cognitive function in hyperammonemic rats. Moreover, enhanced activation of Src leads to increased CCL2, which activates CCR2 in microglia leading to microglial activation. The activation of the S1PR2 → IL-1β → IL-1R → Src → GLUN2B →p38 pathway also leads to increased BDNF levels in neurons. BDNF is released and activates TrkB in astrocytes and microglia, inducing astrocytes activation and sustained microglia activation. Moreover, BDNF also activates TrkB in neurons, leading to activation of the TrkB → PI3K → Akt → CREB pathway, which further increases BDNF levels, thus resulting in sustained neuroinflammation.

We then analyzed in detail the steps involved in the neuroinflammatory process induced by the enhanced activation of S1PR2. We recently showed that, in the cerebellum, the enhanced activation of S1PR2 increases CCL2 levels, which activates CCR2 in microglia leading to microglial activation and enhanced BDNF levels [31]. We hypothesized that a similar pathway would be involved in microglia activation in the hippocampus. We propose the pathway, which is summarized in Figure 5, for the hippocampus of hyperammonemic rats.

We hypothesized that the enhanced activation of S1PR2 would increase IL-1β levels and lead to the activation of its receptor, IL-1R, which leads to the activation of the protein kinase Src, which would mediate the increase in CCL2.

To assess if this hypothesis is correct, we analyzed the effects of hyperammonemia and of blocking S1PR2 or IL-1R on the phosphorylation of Src and CCL2 contents.

**Figure 6:**
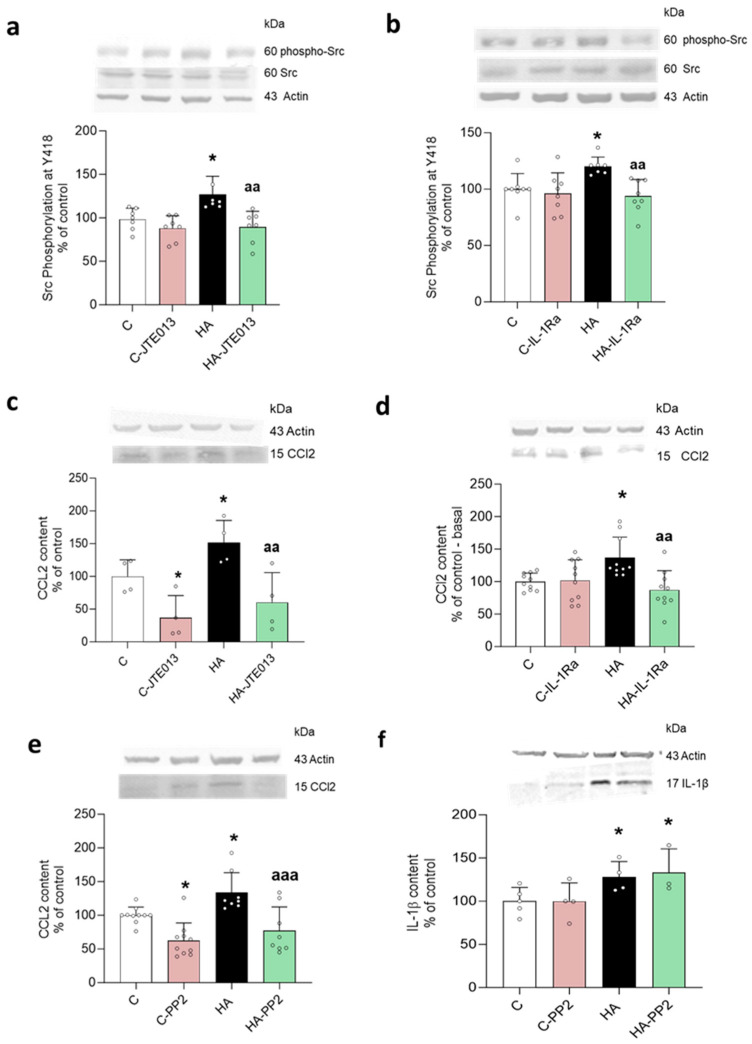
Phosphorylation of Src and CCL2 contents increased in hyperammonemic rats and reversed by blocking S1PR2 and IL-1 receptor with JTE-013 and IL-1Ra, respectively. The increase in CCL2 was also reversed by inhibiting Src with PP2. The phosphorylation of Src at Y418 ((**a**,**b**) *n* = 7–8) and CCL2 contents (**c**–**e**) was analyzed using Western blot in slices of hippocampus from control and hyperammonemic rats treated or not with JTE013 ((**a**,**c**) *n* = 4)), IL-1Ra ((**b**,**d**) *n* = 10), or PP2 ((**e**,**f**) *n* = 8–10 and *n* = 3–5, respectively). The values are the means ± SD of the indicated number of experiments with duplicate samples in each one. The data were analyzed using two-way ANOVA followed by Fisher’s LSD ((**c**) and (**f**)) or Tukey’s ((**a**–**e**)) post hoc test. Values significantly different from the control rats are indicated by asterisks, where * *p* < 0.05, and from the hyperammonemic rats by “a”, where aa *p* < 0.01 and aaa *p* < 0.001.

Hyperammonemia increased the phosphorylation of Src at Y418, which was reversed by blocking S1PR2 with JTE-013 (Figure 6a) or IL-1R with an endogenous antagonist (Figure 6b). Hyperammonemia also increased the CCL2 content in the hippocampus, which was also reversed by blocking S1PR2 (Figure 6c) or IL-1R (Figure 6d).

To assess if the enhanced content of CCL2 was mediated by the increase in Src activity, we tested if inhibiting Src with PP2 reversed the increase in CCL2. As shown in Figure 6e, this was the case. Moreover, inhibiting Src with PP2 did not reduce the increase in IL-1β (Figure 6f). These data indicate that enhanced activation of S1PR2 in the hippocampus of hyperammonemic rats induced the increase in IL-1β content, which activated its receptor, IL-1R, leading to increased phosphorylation and activity of Src, mediating the increase in CCL2.

### 2.5. Increased CCL2 Enhances Activation of CCR2, Leading to Microglia Activation, Increased p38 Phosphorylation and BDNF Levels, Which Activate Astrocytes

We recently showed that increased CCL2 in the cerebellum of hyperammonemic rats activates CCR2 in microglia, leading to microglial activation and enhanced BDNF levels [31]. We assessed if a similar process occurs in the hippocampus.

**Figure 7:**
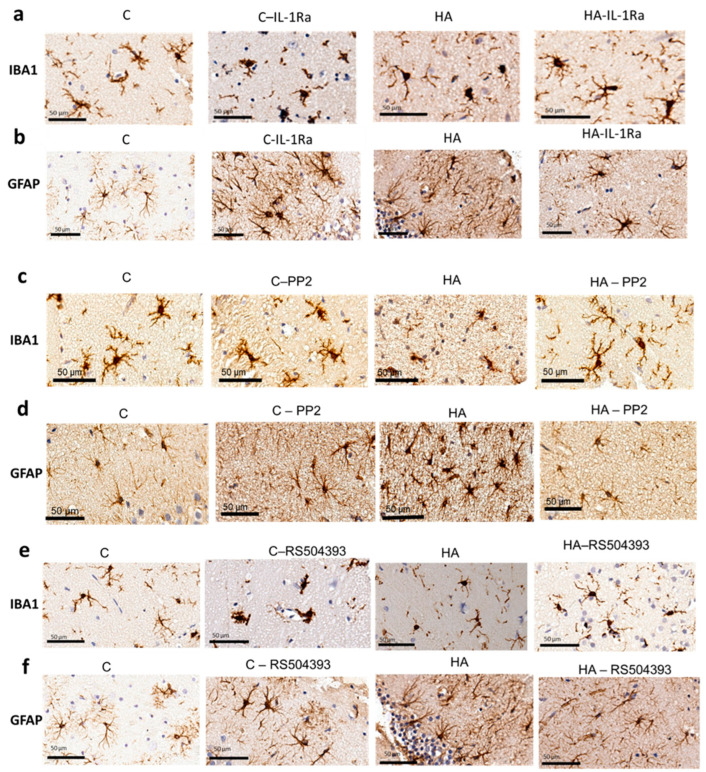

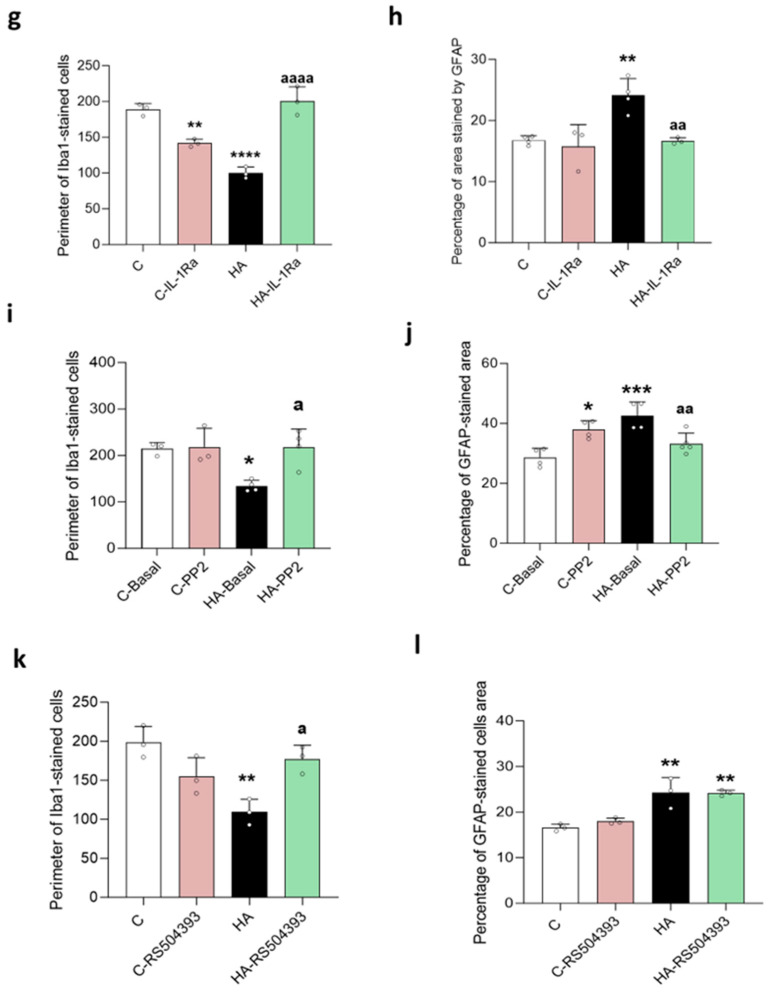
Blocking IL-1R or inhibiting Src reduces microglia and astrocyte activation in the hippocampus of hyperammonemic rats. Blocking CCR2 reduced microglia but not astrocyte activation. Immunohistochemistry was performed on hippocampal slices from the control and hyperammonemic rats treated or not with IL-1Ra (**a**,**b** and **g**,**h**), PP2 (**c**,**d** and **i**,**j**) or RS504393 (**e**,**f** and **k**,**l**) with DAB staining using antibodies against Iba1 (**a**,**c**,**e**) and GFAP (**b**,**d**,**f**). To analyze the microglia activation, the perimeter of the cell was measured. The GFAP-stained area was measured as an indicator of the astrocytes activation. The values are the means ± SD of 3–5 experiments with duplicate samples in each one. The data were analyzed using two-way ANOVA followed by Tukey’s post hoc test. Values significantly different from the control rats are indicated by asterisks, where * *p* < 0.05, ** *p* < 0.01, *** *p* < 0.001, and **** *p* < 0.0001, and from the hyperammonemic rats by “a”, where a *p* < 0.05, aa *p* < 0.01, and aaaa *p* < 0.0001.

We then assessed if activation of IL-1R, Src and CCR2 also mediate the activation of microglia and astrocytes. Blocking IL-1R with an endogenous antagonist reverses the activation of microglia (Figure 7a,g) and astrocytes (Figure 7b,h). The same effects were induced by inhibiting Src with PP2, which reversed the activation of microglia (Figure 7c,i) and astrocytes (Figure 7d,j). Blocking CCR2 with RS504393 also reversed the activation of microglia (Figure 7e,k) but not astrocytes (Figure 7f,l).

These results support the sequences summarized in Figure 5:

(a) S1PR2 → IL-1β → IL-1R → Src → CCL2 → CCR2 → microglia activation.
(b) S1PR2 → IL-1β → IL-1R → Src → astrocytes activation.

In hippocampal neurons, increased levels of IL-1β and the activation of IL-1R and Src increase the phosphorylation of the GLUN2B subunit of the NMDA receptor at Tyr1472, leading to increased entry of Ca^2+^ and the phosphorylation and activation of MAP kinase p38 [19]. The activation of MAP kinase p38 enhances the expression of BDNF [33].

**Figure 8:**
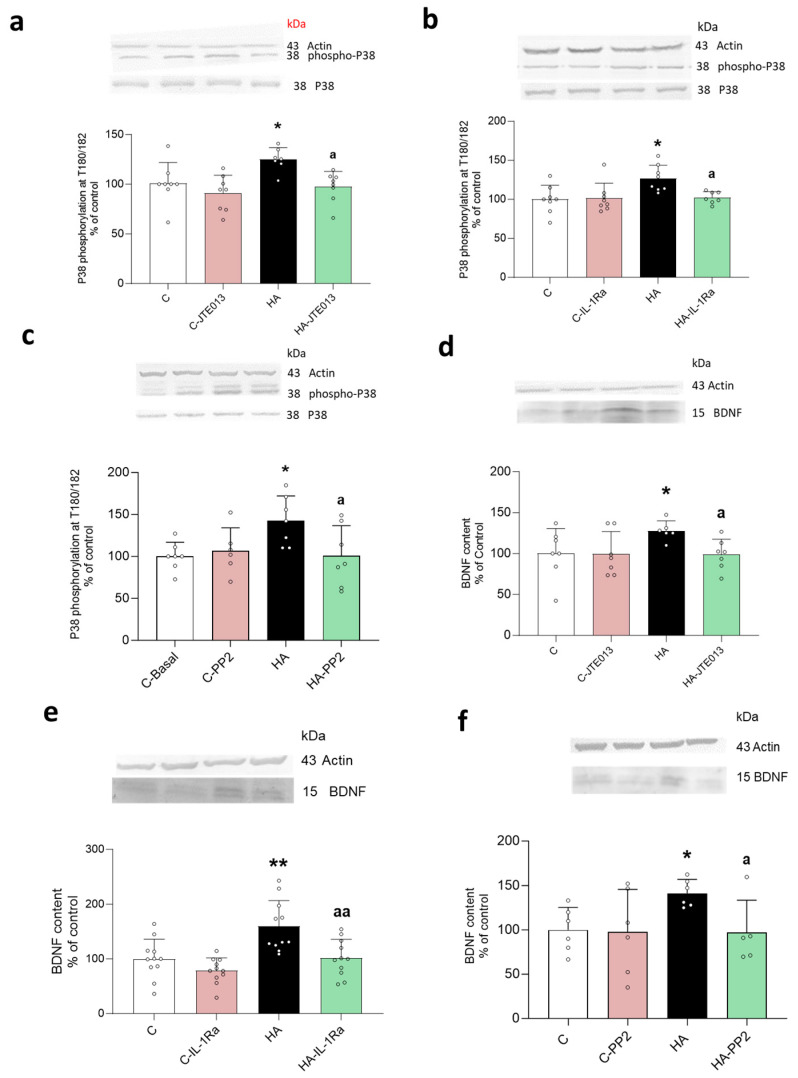
P38-MAPK phosphorylation and BDNF contents increased in the hippocampus of hyperammonemic rats and were reversed by blocking S1PR2 or IL-1R or by inhibiting Src. Western blots were performed in hippocampal slices of control and hyperammonemic rats treated or not with JTE013 ((**a**) *n* = 7–8, (**d**) *n* = 6–7), IL-1Ra ((**b**) *n* = 7–8, (**e**) *n* = 11) or PP2 ((**c**) *n* = 6–7, (**f**) *n* = 5–6). Phosphorylation of MAPK-p38 was analyzed using a specific antibody against p38 phosphorylated at the residue T180/182 (**a**–**c**). The total content of BDNF is shown in (**d**–**f**). The values are the means ± SD of the indicated number of experiments with duplicate samples in each one. The data were analyzed using two-way ANOVA followed by Tukey’s post hoc test (**a**–**c**,**e**) and by Fisher’s LSD post hoc test (**d**,**f**). Values significantly different from the control rats are indicated by asterisks, where * *p* < 0.05 and ** *p* < 0.01, and from the hyperammonemic rats by “a”, where a *p* < 0.05 and aa *p* < 0.01.

We assessed if hyperammonemia increases the phosphorylation of p38 and the content of BDNF and if these effects are mediated by the S1PR2 → IL-1β → IL-1R → Src pathway. Hyperammonemia increased the phosphorylation of p38 MAP kinase at T180/182, which was reversed by blocking S1PR2 (Figure 8a) or IL-1R (Figure 8b) or by inhibiting Src with PP2 (Figure 8c).

Hyperammonemia also increased the BDNF content in the hippocampus as analyzed using Western blot, and this increase was reversed by blocking S1PR2 (Figure 8d) or IL-1R (Figure 8e) or by inhibiting Src (Figure 8f).

### 2.6. BDNF Levels Increased in CA1 Neurons, Leading to Increased Activation of TrkB, Which Contributes to a Sustained Increased in BDNF and Activation of Microglia and Astrocytes

**Figure 9:**
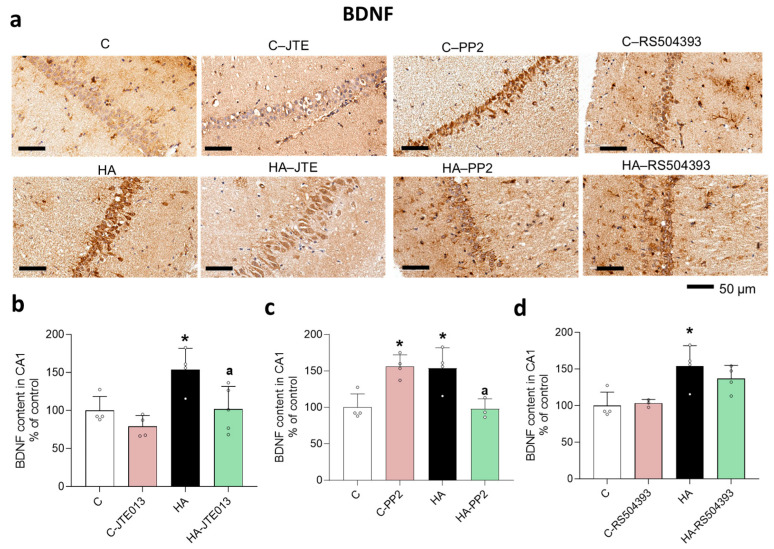
BDNF increased in neurons of the CA1 region of the hippocampus of hyperammonemic rats. This increase was reversed by blocking S1PR2 or inhibiting Src but not by blocking CCR2. Immunohistochemistry was performed on hippocampal slices from control and hyperammonemic rats treated or not with JTE013, PP2 or RS504393 using antibodies against BDNF (**a**). Quantification of BDNF in CA1 is shown in (**b**–**d**) as percentage of the controls. The values are the means ± SD of 4–5 experiments (**b**) and 3–4 experiments (**c**,**d**) with duplicate samples in each one. The data were analyzed using two-way ANOVA followed by Tukey’s post hoc test. Values significantly different from control rats are indicated by asterisks, where * *p* < 0.05, and from the hyperammonemic rats indicated by “a”, where a *p* < 0.05.

We assessed, by immunohistochemistry, if the increase in BDNF occurred in CA1 neurons. As shown in Figure 9, BDNF increased in neurons of the CA1 region of the hippocampus of hyperammonemic rats. The increase in BDNF was reversed by blocking S1PR2 with JTE-013 (Figure 9a,b) or by inhibiting Src with PP2 (Figure 9a,c) but not by blocking CCR2 with RS504393 (Figure 9a,d).

**Figure 10:**
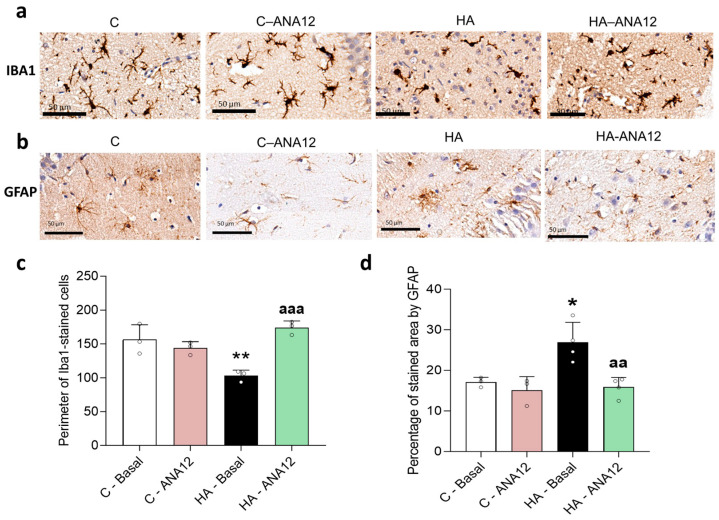
Blocking the BDNF receptor TrkB reduced the microglia and astrocyte activation in the hippocampus of hyperammonemic rats. Immunohistochemistry was performed on hippocampal slices from control and hyperammonemic rats treated or not with ANA12 using antibodies against Iba1 (**a**,**c**) and GFAP (**b**,**d**). To analyze the microglia activation, the perimeter of the cell was measured. The GFAP-stained area was measured as an indicator of the astrocytes activation. The values are the means ± SD of 3–4 experiments with duplicate samples in each one. The data were analyzed using two-way ANOVA followed by Tukey’s post hoc test. Values significantly different from the control rats are indicated by asterisks, where * *p* < 0.05 and ** *p* < 0.01, and from the hyperammonemic rats by “a”, where aa *p* < 0.01 and aaa *p* < 0.001.

Increased levels of BDNF may contribute to the sustained activation of microglia and astrocytes [34]. To assess this possibility, we analyzed the effects of blocking the BDNF receptor TrkB with ANA12. As shown in Figure 10, blocking TrkB reversed the activation of the microglia (Figure 10a,c) and astrocytes (Figure 10b,d).

**Figure 11:**
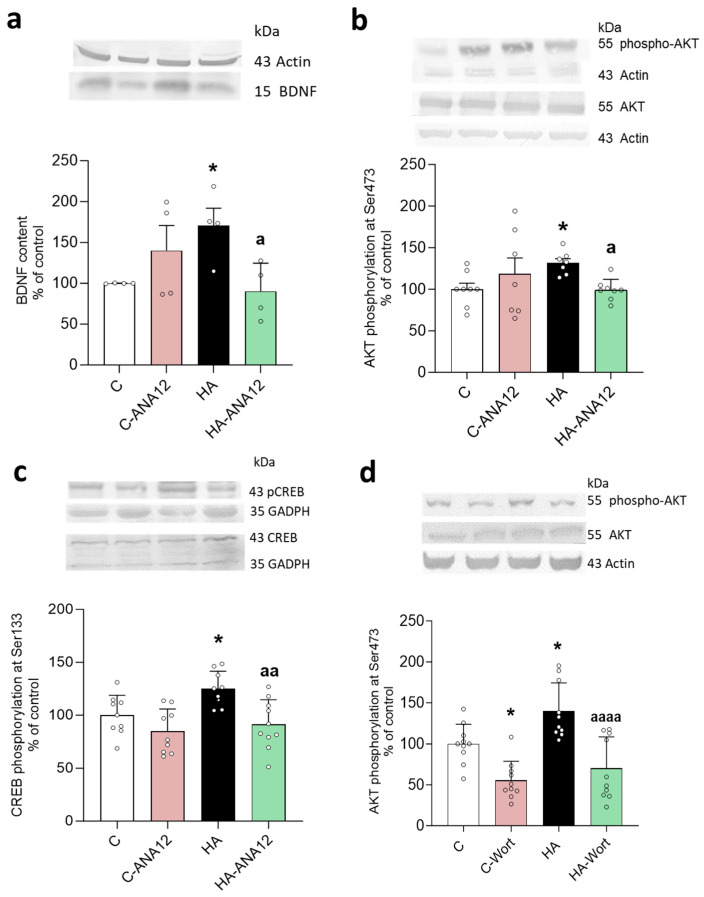

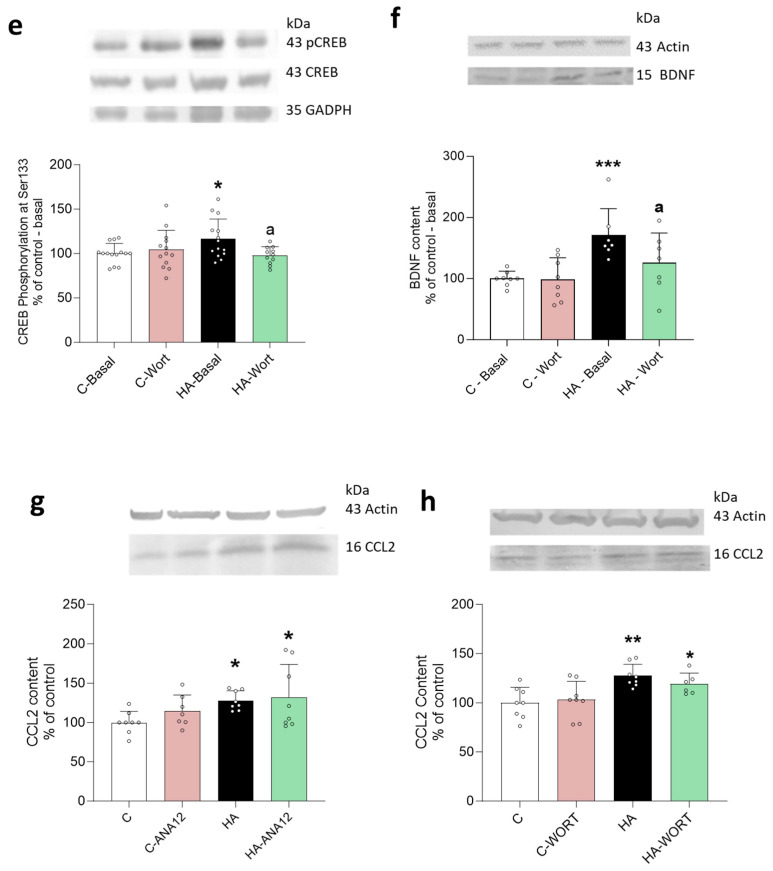
Blocking the BDNF receptor TrkB reduced the BDNF content and AKT and CREB phosphorylation. The inhibition of PI3K with wortmannin reduced AKT and CREB phosphorylation and BDNF content in the hippocampus of hyperammonemic rats. Western blots were performed on hippocampal slices of control and hyperammonemic rats treated or not with ANA 12 (**a**–**c**,**g**) or wortmannin to inhibit PI3K (**d**–**f**,**h**). The phosphorylation of AKT was analyzed using a specific antibody against AKT phosphorylated at S473 ((**b**) *n* = 7–8, (**d**) *n* = 10). The phosphorylation of CREB was analyzed using a specific antibody against CREB phosphorylated at S133 ((**c**) *n* = 9–10, (**e**) *n* = 11–12). The total content of BDNF is shown in (**a**) (*n* = 4) and (**f**) (*n* = 7–8) and of CCL2 in (**g**) (*n* = 7–8) and (**h**) (*n* = 6–8). The values are the means ± SD of the indicated number of experiments with duplicate samples in each one. The data were analyzed using two-way ANOVA followed by Tukey’s post hoc (**c**–**e**) and Fisher’s LSD post hoc tests (**a**,**b**,**f**–**h**). Values significantly different from the control rats are indicated by asterisks, where * *p* < 0.05, ** *p* < 0.01, and *** *p* < 0.001, and from the hyperammonemic rats by “a”, where a *p* < 0.05, aa *p* < 0.01, and aaaa *p* < 0.0001.

Moreover, blocking TrkB also reversed the increase in BDNF (Figure 11a). These data suggest that the activation of astrocytes requires the activation of TrkB by BDNF, while the initial microglial activation was mediated by the activation of CCR2, but the maintenance of a sustained microglia activation would require the activation of TrkB by BDNF. Moreover, the activation of TrkB by BDNF would also contribute to a sustained elevation of BDNF.

### 2.7. Enhanced Activation of TrkB-PI3K-Akt-CREB Pathway Contributes to Enhanced Levels of BDNF but Not CCL2 in Hippocampus of Hyperammonemic Rats

The activation of TrkB may enhance BDNF levels by activating the PI3K-Akt-CREB pathway [35,36,37,38]. We found that hyperammonemia increases Akt activation, as indicated by the increased phosphorylation of Akt at Ser473 (Figure 11b), and CREB activity, as indicated by its increased phosphorylation at Ser133 (Figure 11c). Both AKT and CREB phosphorylation were reduced to normal levels by blocking TrkB with ANA12 (Figure 11b,d) or by inhibiting PI3K with wortmannin (Figure 11d,e). Wortmannin also reverses the increase in BDNF in hyperammonemic rats (Figure 11f).

This TrkB-PI3K-Akt-CREB pathway was not involved in the increase in CCL2 in the hyperammonemic rats, as supported by the fact that blocking TrkB with ANA12 (Figure 11g) or inhibiting PI3K with wortmannin (Figure 11h) did not reverse the increase in CCL2.

## 3. Discussion

The results reported unveil key pathways involved in the induction and maintenance of neuroinflammation in the hippocampus and cognitive impairment in chronic hyperammonemia. Moreover, these data provide a new therapeutic approach to improving cognitive function in hyperammonemic rats by blocking these pathways through the inhibition of an initial step in the process: blocking S1PR2 activation using a selective antagonist, JTE-013.

These data support that chronic hyperammonemia increases the function of the pathways in the hippocampus, as summarized in Figure 5:

(a) S1PR2 → IL-1β → IL-1R → Src → GLUN2B → p38 → altered membrane expression of AMPA receptors → impaired cognitive function;
(b) S1PR2 → IL-1β → IL-1R → Src → CCL2 → CCR2 → microglia activation;
(c) S1PR2 → IL-1β → IL-1R → Src → GLUN2B → p38 → BDNF → TrkB → astrocytes activation;
(d) S1PR2 → IL-1β → IL-1R → Src → GLUN2B →p38 → BDNF → TrkB → PI3K → Akt → CREB → BDNF.

An initial event is the enhanced activation of S1PR2 due to the enhanced membrane expression of the receptor and increased content of its agonist S1P. The enhanced activation of S1PR2 increases the IL-1β content and activation of its receptor and increases the phosphorylation and activity of the Src protein kinase. The activation of Src in hippocampal neurons by IL-1β has also been reported by Ghosh et al. [39,40,41].

The enhanced activation of Src mediates the increase in the membrane expression of the GLUN2B subunit of NMDA receptors, [19,42,43], which results in increased phosphorylation and activity of MAP kinase p38 and altered membrane expression of GluA1 and GluA2 subunits of AMPA receptors, which leads to cognitive impairment, which is in agreement with the pathway described by Taoro-Gonzalez et al. [19]. Blocking S1PR2 with JTE-013 in hyperammonemic rats in vivo reverses this entire pathway and restores cognitive function. This supports that S1PR2 is a new therapeutic target to restore cognitive function in hyperammonemia and in patients with hepatic encephalopathy.

Moreover, the activation of S1PR2 also contributes to maintaining neuroinflammation in the hippocampus via different mechanisms. The activation of the S1PR2 → IL-1β → IL-1R → Src pathway also increases CCL2, which activates CCR2 in microglia leading to microglial activation. The activation of microglia by CCL2 has also been reported in different pathological situations associated with neuroinflammation and cognitive impairment [44,45,46]. This pathway mediates the initial activation of microglia, but sustained activation would also require a sustained increase in BDNF levels, as indicated by the fact that blocking the BDNF receptor TrkB also reverses microglia activation.

A role for BDNF In promoting microglia and astrocytes activation and neuroinflammation has been reported in other pathological situations. Ding et al. [34] showed that BDNF promotes the activation of astrocytes and microglia contributing to neuroinflammation and mechanical allodynia in cyclophosphamide-induced cystitis. Astrocytes express high levels of TrkB, which modulate morphological maturation, as well as astrocytes activation [47]. We showed here that astrocytes activation in the hippocampus of hyperammonemic rats is mediated by S1PR2 → IL-1β → IL-1R → Src → p38 → BDNF → TrkB → astrocytes activation. An enhancement in BDNF production in rat hippocampus by the activation of p38 has also been reported [48]. The activation of astrocytes by neuronal BDNF has also been reported by Jeon et al. [49].

Finally, we also showed that the hippocampus of hyperammonemic rats enhanced the function of the BDNF → TrkB → PI3K → Akt → CREB → BDNF pathway, which contributes to maintaining the production of BDNF and sustaining glial activation and neuroinflammation. A similar reduction in BDNF levels, induced by blocking TrkB with ANA12, has been reported by Ding et al. [34] in the spinal cord. They suggest that a reduction in BDNF is due to reduced TNFα levels and activation of TNFR1. However, we showed here that wortmannin also reduces BDNF levels, supporting that a reduction in BDNF levels by ANA12 in the hippocampus of hyperammonemic rats was due to the reduced activation of the TrkB → PI3K → Akt → CREB→ BDNF pathway. A similar increase in BDNF levels through activation of the PI3K-Akt-CREB pathway has previously been reported [34,35,36,37,38]. The enhanced activation of the TrkB → PI3K → Akt → CREB→ BDNF pathway in the hippocampus of hyperammonemic rats contributes to the sustained maintenance of increased BDNF levels, which, in turn, contributes to the sustained activation of microglia and astrocytes and neuroinflammation.

Sphingolipids are ubiquitously present in eukaryotic cell membranes and act as signaling molecules in the regulation of many cellular processes [50,51,52].

Recent reports suggest a key role of sphingolipids in diseases associated with neuroinflammation, including Alzheimer’s and Parkinson’s diseases and a potential of sphingolipids as a new therapeutic and diagnostic target for these diseases [53,54,55,56]. S1P is a key sphingolipid which modulates neuroinflammation through its receptors S1PR1 and S1PR2 [26,57,58]. Fingolimod is an agonist of several S1P receptor subtypes except for S1P2R, which has been approved as a treatment for relapsing–remitting multiple sclerosis and has been proposed as being useful in other neurological disorders such as Parkinson’s disease [59,60]. 

However, the effects of fingolimod are not mediated by S1PR2, which is not modulated by this drug.

A few reports suggest a beneficial effect of reducing S1PR2 activation to reduce neuroinflammation. McMillin et al. [26] reported that enhanced activation of S1PR2 promotes neuroinflammation in the cerebral cortex of mice with hepatic encephalopathy induced by azoxymethane [26]. Kim et al. [61] showed that complement component 8 gamma (C8G) reduces neuroinflammation and blood–brain barrier permeability and inhibits glial hyperactivation and cognitive decline in acute and chronic animal models of Alzheimer’s disease by antagonizing S1PR2 activation. The enhanced activation of S1PR2 also induces neuroinflammation and enhances GABAergic neurotransmission in the cerebellum of hyperammonemic rats, leading to motor incoordination, which is reversed by blocking S1PR2 with JTE-013 [31].

We showed here that blocking S1PR2 also reduces neuroinflammation and restores glutamatergic neurotransmission in hippocampus and cognitive function in hyperammonemic rats. These reports support the idea that blocking S1PR2 may have beneficial effects on neuroinflammation and cognitive and motor function in different pathologies, including hyperammonemia, hepatic encephalopathy and Alzheimer’s disease.

### Limitations of the Study and Future Research Directions

A limitation of the study is that the administration of the antagonist of the S1PR2 in vivo to assess the effects on cognitive function has been performed using intracerebral administration with mini-osmotic pumps. This was necessary for technical and economic reasons. It would be desirable to have an antagonist of S1PR2 that could be administered orally or by injection at a reasonable cost. Future research should assess the utility of S1PR2 antagonists in human patients. The results reported here and in [31] support that these compounds would have beneficial effects to treat cognitive and motor impairment in cirrhotic patients with minimal hepatic encephalopathy. It is likely that they may also be beneficial in other pathological situations associated with sustained neuroinflammation.

## 4. Materials and Methods

The animals were male Wistar rats, which became hyperammonemic by feeding them an ammonium-containing diet, as described in [16]. The experiments were approved by the Comite de Etica y Experimentación Animal (Principe Felipe Research Center) and by Generalitat Valenciana, and which were performed in accordance with the European Communities Council Directive 2010/63/EU.

### 4.1. In Vivo Experiments

#### 4.1.1. Continuous Intracerebral Administration of JTE-013 to Rats Using Osmotic Pumps

The following groups were used:

(1) CV: Control rats implanted with mini-osmotic pumps containing vehicle (sterile saline + 1% DMSO).
(2) CJ: Control rats implanted with mini-osmotic pumps containing 1.22 mM JTE-013 (383150-41-2, Axon Medchem BV, Groningen, The Netherlands) diluted in sterile saline + DMSO 1%, as in Kimura et al. [62].
(3) HV: hyperammonemic rats implanted with mini-osmotic pumps containing vehicle.
(4) HJ: Hyperammonemic rats implanted with mini-osmotic pumps containing JTE-013.

The pumps were implanted 2 weeks after beginning the ammonia-containing diet. These pumps released 0.25 µL per hour for 28 days and were connected to a cannula (Brain infusion kit 2, 3–5 mm, ALZET, Cupertino, CA, USA) implanted in the cerebral ventricle, under isoflurane anesthesia, as in [18]

#### 4.1.2. Evaluation of Spatial Learning in the 8-Arms Radial Maze

The rats were habituated to the maze, and the test was performed over the following 4 days, with 5 trials per day, as in [14]. The number of reference memory errors, working memory errors and the learning index were calculated, as in [14].

#### 4.1.3. Evaluation of Novel Object Recognition (NOR) Memory

The NOR memory test was performed, as in Taoro-Gonzalez et al. [19], in an open-field arena (70 × 70 × 40 cm) of black painted wood with visuospatial cues on the walls. The rats were habituated over 4 days in 2 sessions of 5 min per day, allowing them to explore the empty arena. The NOR test was performed on day 5. During the sample phase, 2 identical objects were placed in the cage and the rat was allowed to explore them for 3 min. The test phase was performed after 6 h, with the objects located in the same position but changing one of the objects for an unexplored one and allowing the rat to freely explore again for 3 min. Sessions were recorded with a digital camera and the exploration time of the familiar stimulus and the novel stimulus (i.e., unexplored object) was counted. The discrimination ratio test was calculated as the following: ((time exploring novel stimulus − time exploring familiar stimulus)/total exploration time).

### 4.2. Ex Vivo Experiments

The rats were sacrificed by decapitation at 4–5 weeks of hyperammonemia and their brains quickly transferred to a plate. Hippocampal slices were perfused with Krebs buffer (in mM: NaCl 119, KCl 2.5, KH_2_PO_4_ 1, NaHCO_3_ 26.2, CaCl_2_ 2.5 and glucose 11, aerated with 95% O_2_ and 5% CO_2_ at pH 7.4) (basal) or with different treatments in Krebs buffer for 30 min. The treatments were as follows: JTE-013 20 μM, an antagonist of S1PR2 (2392; Tocris/Bio-Techne, Minneapolis, MN, USA); IL-1Ra 100 ng/mL (1545-RA-025; R&D Systems, Minneapolis, MN, USA); ANA12 50 μM, an antagonist of TrkB (478; Tocris/Bio-Techne, Minneapolis, MN, USA); RS504393 50 μM a CCR2 antagonist (2517; Tocris/Bio-Techne, Minneapolis, MN, USA); PP2 at 10 μM, an inhibitor of Src (P0042; Sigma, Darmstadt, Germany); or wortmannin 2 μM, a PI3K inhibitor (12-338; Sigma, Darmstadt, Germany).

#### 4.2.1. Analysis of Protein Content and Phosphorylation in Hippocampal Slices Using Western Blotting

Hippocampal slices incubated or not with different treatments, as above, were homogenized by sonication for 20 s in a buffer (Tris-HCl 66 mM pH 7.4, SDS 1%, EGTA 1 mM, glycerol 10%, leupeptin 0.2 mg/mL, NaF 1 mM, Na orto-vanadate 1 mM) and were subjected to immunoblotting. Primary antibodies against IL-1β (1:1000; AF-501-NA; R&D SISTEMS, Minneapolis, MN, USA), CCl2 (1:1000; 66272-1-lg; Proteintech, Manchester, UK), BDNF (1:1000; OSB00017W; Invitrogen, Waltham, MA, USA), Src phosphorylated at tyrosine 418 (1:1000; ab40660; Abcam, Cambridge, UK) and CREB phosphorylated at serine 133 (1:1000; 06-519; UPSTATE, Sigma, St. Louis, MO, USA) were used. Phosphorylation levels were normalized to the total amount of the corresponding protein. As a control for protein loading, the same membranes used to quantify the amount of proteins were incubated with an antibody against Actin (1:5000; ab6276; Abcam) or GAPDH (1:4000; MAB374; Millipore, Burlington, MA, USA) depending on the molecular mass of the proteins. Secondary antibodies were anti-rabbit (cat# A8025), anti-goat (cat# A7650) or anti-mouse (cat# A3562) IgG with a 1:4000 dilution conjugated with alkaline phosphatase from Sigma. The images were captured using the ScanJet 5300C (Hewlett-Packard, Amsterdam, The Netherlands), and the band intensities were quantified using the Alpha Imager 2200, version 3.1.2 (AlphaInnotech Corporation, San Francisco, CA, USA).

#### 4.2.2. Analysis of Membrane Expression of AMPA and NMDA Receptor Subunits and S1PR2 

The membrane expression was analyzed, as previously described by Cabrera-Pastor et al. [18], by cross-linking with bis(sulfosuccinimidyl)suberate (BS3; 21580; Pierce, Rockford, IL, USA). After the treatments, the slices were placed in tubes containing ice-cold Krebs buffer with or without 2 mM BS3, as in [18]. Samples were subjected to Western blotting to analyze the membrane expression of GluN2A (Millipore 04-901) and GluN2B (06-600, Millipore) NMDA receptor subunits, GluA1 (PC246, Calbiochem, Merck KGaA, Darmstadt, Germany) and GluA2 (AB1768-I Millipore) AMPA receptor subunits and S1PR2 (21180-1-AP, Proteintech). The secondary antibodies were anti-rabbit (1:4000; A3687) or anti-mouse (1:4000, A3562) from Sigma.

#### 4.2.3. Immunohistochemistry

After the incubation of the hippocampal slices with different treatments, as described above, the slices were fixed in 4% paraformaldehyde in 0.1 M phosphate buffer (pH 7.4) for 24 h at 4 °C. Paraffin-embedded slices were cut and mounted on a coated slide glass, processed with the Envision Flex + kit (DAKO) blocking endogenous peroxidase activity for 5 min and incubated with antibodies against Iba1 (1:400; 019-19741; FUJIFILM Wako Chemicals USA Corp. Richmond, VA, USA), GFAP (1:400; G-3893; Sigma) or BDNF (1:200; Ab6201; Abcam) for 30 min. The reaction was visualized using Envision Flex + horseradish peroxidase for 20 min and, finally, diaminobenzidine for 10 min. The sections were counterstained with Mayer’s hematoxylin for 5 min.

The Analysis of ionized calcium-binding adapter molecule 1 (Iba1) staining was performed using the software Image Pro Plus version 6.0 IP-Win 32. Hippocampal slices from five–six animals per group were used. Microglial activation was analyzed by measuring the perimeter of Iba1-stained cells in 10 randomly selected 56x fields per section. The results are expressed in micrometers. For astrocytes activation we quantified the area stained with GFAP using ImageJ version 1.8.0 software. For each rat, at least 10 56× fiel-ds were quantified. The results are expressed as the percentage of the area stained with GFAP.

The BDNF content of the neurons of the CA1 region was analyzed using the ROI manager function in ImageJ software. Neurons of the CA1 region were manually outlined and the mean intensity was measured. The results are expressed as a percentage of the control group.

### 4.3. Statistical Analysis

All statistical analyses were performed using the software program GraphPad Prism version 8.0. The results are expressed as the mean ± standard error. The number of samples necessary to analyze the membrane expression, content or phosphorylation of the different proteins using Western blot was adjusted in each case to reduce the variability. The normality distribution was assessed using D’Agostino and Pearson, Shapiro–Wilk and Kolmogorov–Smirnov normality tests. The differences in the variances of normally distributed data were assessed using Bartlett’s test. Data with the same variance across groups were analyzed using a parametric two-way analysis of variance (ANOVA) followed by Tukey’s or Fisher’s LSD post hoc tests. Data with different variances across groups were analyzed using Kruskal–Wallis, a nonparametric test, followed by Dunnett’s post hoc test. A confidence level of 95% was accepted as significant. The number of animals used for each parameter is indicated in the corresponding figure legend.

## 5. Conclusions

This report unveils key pathways involved in the induction and maintenance of neuroinflammation in the hippocampus and cognitive impairment in chronic hyperammonemia (Figure 5). Hyperammonemia increases S1PR2 activation in the hippocampus by increasing the level of S1P and membrane expression of S1PR2. This increases IL-1β in neurons and activation of its receptor IL-1R, which enhances Src activity leading to increased phosphorylation and membrane expression of the GLUN2B subunit of NMDA receptors. This leads to increased activity of p38 MAP kinase and altered membrane expression of the GluA1 and GluA2 subunits of AMPA receptors, which results in cognitive impairment. Blocking S1PR2 with JTE-013 in vivo restores cognitive function in hyperammonemic rats. Moreover, enhanced activation of Src leads to increased CCL2, which activates CCR2 in microglia leading to microglial activation. The activation of the S1PR2 → IL-1β → IL-1R → Src → GLUN2B →p38 pathway also leads to increased BDNF levels in neurons. BDNF is released and activates TrkB in astrocytes and microglia, inducing astrocytes activation and sustained microglia activation. Moreover, BDNF also activates TrkB in neurons, leading to activation of the TrkB → PI3K → Akt → CREB pathway, which further increases BDNF levels, thus resulting in sustained neuroinflammation.

These data provide a new therapeutic approach to improve cognitive function by blocking these pathways at any step. We show that inhibiting an initial step of the process—the activation of S1PR2—using a selective antagonist, JTE-013, restores cognitive function in hyperammonemic rats. Reducing S1PR2 activation may be also useful for improving cognitive function in patients with liver disease and hepatic encephalopathy and, likely, in other pathologies associated with neuroinflammation.

## Data Availability

The data presented in this study are available on request from the corresponding author.
